# Bioinformatics analysis of signature genes related to cell death in keratoconus

**DOI:** 10.1038/s41598-024-63109-5

**Published:** 2024-06-03

**Authors:** Jinghua Liu, Juan Gao, Shulei Xing, Yarong Yan, Xinlin Yan, Yapeng Jing, Xuan Li

**Affiliations:** 1 School of Medicine, Nankai University, Tianjin, 300071 China; 2 Nankai University Affiliated Eye Hospital, Tianjin, 300020 China; 3 Tianjin Eye Hospital, Tianjin Key Lab of Ophthalmology and Vision Science, Tianjin Eye Institute, Tianjin, 300020 China; 4 Clinical College of Ophthalmology, Tianjin Medical University, Tianjin, 300020 China

**Keywords:** Bioinformatics, Diagnostic markers

## Abstract

Keratoconus is corneal disease in which the progression of conical dilation of cornea leads to reduced visual acuity and even corneal perforation. However, the etiology mechanism of keratoconus is still unclear. This study aims to identify the signature genes related to cell death in keratoconus and examine the function of these genes. A dataset of keratoconus from the GEO database was analysed to identify the differentially expressed genes (DEGs). A total of 3558 DEGs were screened from GSE151631. The results of Gene Ontology (GO) and Kyoto Encyclopedia of Genes and Genomes (KEGG) analysis showed that they mainly involved in response to hypoxia, cell–cell adhesion, and IL-17 signaling pathway. Then, the cell death-related genes datasets were intersected with the above 3558 DEGs to obtain 70 ferroptosis-related DEGs (FDEGs), 32 autophagy-related DEGs (ADEGs), six pyroptosis-related DEGs (PDEGs), four disulfidptosis-related DEGs (DDEGs), and one cuproptosis-related DEGs (CDEGs). After using Least absolute shrinkage and selection operator (LASSO), Random Forest analysis, and receiver operating characteristic (ROC) curve analysis, one ferroptosis-related gene (TNFAIP3) and five autophagy-related genes (CDKN1A, HSPA5, MAPK8IP1, PPP1R15A, and VEGFA) were screened out. The expressions of the above six genes were significantly decreased in keratoconus and the area under the curve (AUC) values of these genes was 0.944, 0.893, 0.797, 0.726, 0.882 and 0.779 respectively. GSEA analysis showed that the above six genes mainly play an important role in allograft rejection, asthma, and circadian rhythm etc. In conclusion, the results of this study suggested that focusing on these genes and autoimmune diseases will be a beneficial perspective for the keratoconus etiology research.

## Introduction

In the early stages, keratoconus presents as uncorrectable myopia and astigmatism. As the conical dilation progresses, visual acuity decreases and corneal perforation may occur. The pathogenesis of keratoconus is believed to involve genetic mechanisms, family history, allergic diseases, and specific diseases, although the exact etiology remains unclear^1,2^. Current treatments for keratoconus include corneal collagen crosslinking, rigid contact lenses, and keratoplasty^3^, but these interventions primarily aim to delay corneal thinning progression. Therefore, understanding the etiology and pathogenesis of keratoconus and developing effective treatments are major research priorities for this disease. Studies have shown that cell apoptosis in the corneal tissue of keratoconus patients is higher compared to normal corneal tissue across all layers^4^. Differential expression of several proteins in the cornea of keratoconus leads to alterations in corneal collagen, cell apoptosis, and necrosis in the stroma, ultimately resulting in changes in the structural integrity of the cornea^5^. To date, 117 proteins and protein classes have been implicated in the pathophysiology of keratoconus^6^. However, further research is needed to elucidate the specific roles of these proteins and protein-coding genes in the pathology of keratoconus.

Autophagy and apoptosis have a reciprocal relationship, whereby they inhibit each other^7^. In normal cellular conditions, autophagy serves a protective function, while apoptosis is responsible for eliminating aging or damaged cells^8^. Autophagy suppresses cell apoptosis by affecting the involved factors to a lesser extent. However, excessive autophagy, leading to the excessive consumption of intracellular proteins and organelles, can trigger cell apoptosis^7,9^. Furthermore, through further investigation, researchers have gained insights into various forms of programmed cell death, such as ferroptosis, cuproptosis, pyroptosis, and disulfidptosis, which are believed to contribute to the development of various diseases^10–12^. As mentioned earlier, cell apoptosis is observed to increase in almost all layers of the cornea in keratoconus. However, it remains unclear whether the etiology and mechanisms underlying the development of keratoconus are associated with the aforementioned cell death-related genes. Unfortunately, due to the lack of well-established animal models for keratoconus, research in this field primarily relies on clinical observations and donor corneal samples. Therefore, based on the available datasets and bioinformatics tool (such as R software), conducting bioinformatics analysis might be one of the most suitable approaches for studying keratoconus, including differential expression analysis, functional enrichment analysis and signature genes analysis.

In this study, our hypothesis was that the etiology and development mechanism of keratoconus are associated with genes related to cell death processes such as autophagy, ferroptosis, cuproptosis, pyroptosis, and disulfidptosis. To investigate this hypothesis, we employed various bioinformatic methods to identify genes involved in autophagy and non-apoptotic cell death in keratoconus. The expression levels of these genes were found to be significantly different between keratoconus and normal cornea, and these findings were further validated using external datasets. Additionally, we conducted an analysis to determine the enrichment of signaling pathways associated with these genes. This study may contribute to a better understanding of the pathogenesis of keratoconus and offer new insights into its diagnosis and treatment.

## Materials and methods

### Data sources

**Figure 1:**
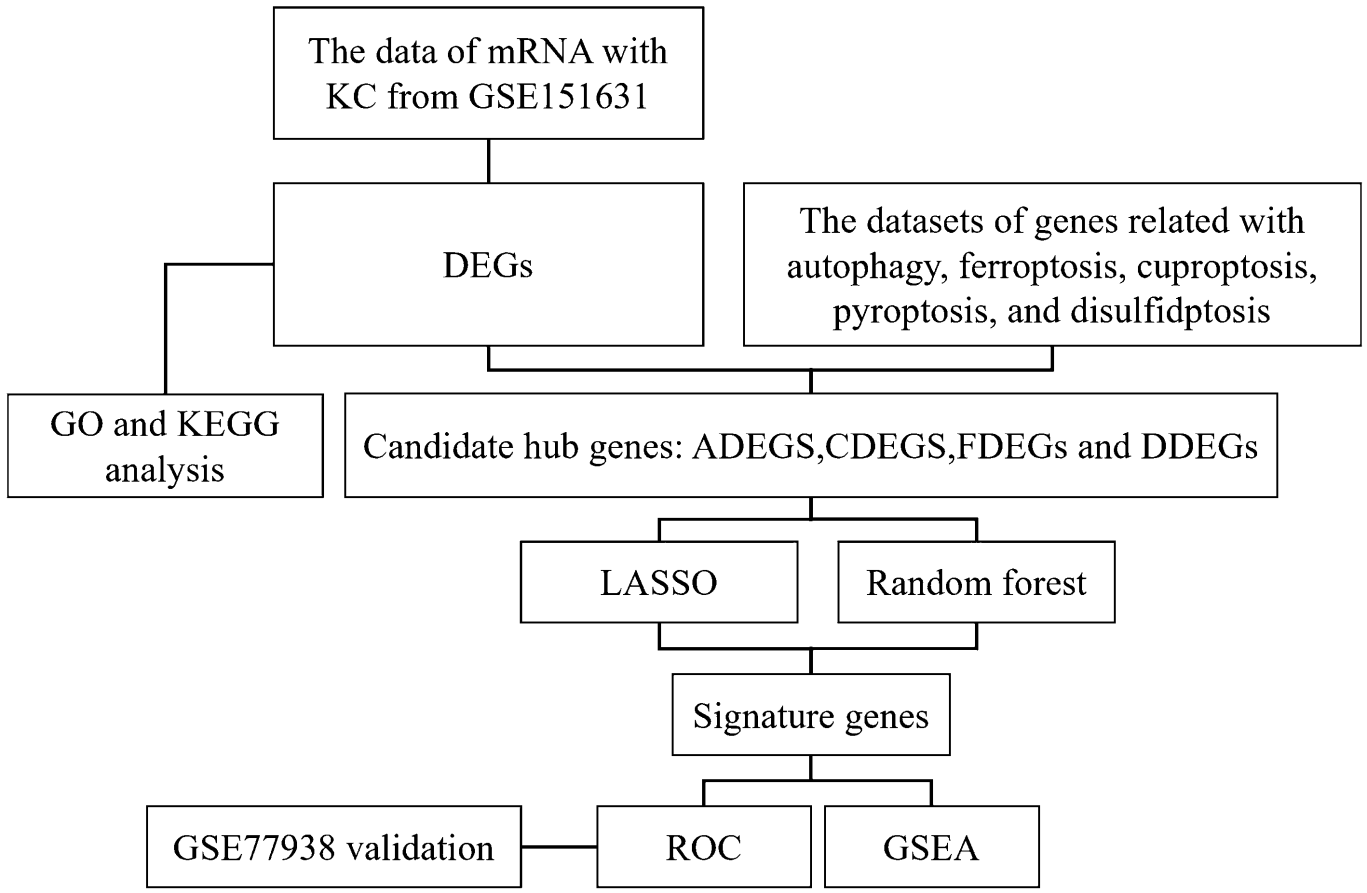
The flow chart of this research.

The keywords “keratoconus” was used to perform a thorough search in the GEO database (https:// www.ncbi.nlm.nih.gov/GEO/) on March 1, 2023. The following criteria of the datasets were included: (1) Transcriptome expression profiles; (2) Participants: patients with keratoconus and the control group; (3) Corneal samples should include corneal epithelium and stroma; (4) Sample size ≥ 20. There were no other specific exclusion criteria. Based on the above criteria, two datasets were included finally. GSE151631 was designated as the training set, comprising 19 individuals with keratoconus and 7 control subjects. GSE77938 was designated as the validation group, consisting of 25 individuals with keratoconus and 25 control subjects. The differential expression analysis, enrichment analysis, signature genes analysis and receiver operating characteristic (ROC) curve analysis were conducted in training set, while ROC curve analysis was conducted in validation set (Fig. 1).

### Identification of DEGs and functional and pathway enrichment analysis

The Gene Count Expression Matrices files of GSE151631 and GSE77938 were downloaded and randomly be divided into training and validation sets. Different sequencing platforms and sample processing methods might lead to different number of genes in the above two datasets. Then, for data preprocessing, we removed genes with low expression. The variance stabilizing transformation (VST), a library discrepancy from DESeq2 package, was applied to correct the matrices^13^. DEGs were analyzed between the keratoconus and control cohorts using the DEseq2 package only for training set. The criterion for DEG selection was set as an adjusted p-value of less than 0.05 and an absolute log2 fold change (logFC) greater than 1. A volcano plot was generated to visualize these DEGs, and a heatmap was used to display the top 50 up-regulated and top 50 down-regulated DEGs.

The functional enrichment analyses of DEGs were conducted using the clusterProfiler package in the R software^14^. The analyses were based on the Gene Ontology (GO) and Kyoto Encyclopedia of Genes and Genomes (KEGG) databases. The GO analysis involved exploring the biological processes of the DEGs through three categories: cellular component (CC), biological process (BP), and molecular function (MF). Furthermore, the KEGG analysis was employed to identify potential signaling pathways.

### Identification of cell death related DEGs and functional and pathway enrichment analysis

Autophagy related genes dataset (http://www.autophagy.lu/), cuproptosis related genes dataset (http://www.zhounan.org/ferrdb/current/operations/download.html), disulfidptosis related genes dataset (http://www.zhounan.org/ferrdb/current/operations/download.html), ferroptosis related genes dataset (http://www.zhounan.org/ferrdb/current/) and the pyroptosis related genes dataset^15^ were took intersections with the DEGs respectively by using the VennDiagram package in the R software^16^. Through this analysis, we identified the autophagy-related DEGs (ADEGs), cuproptosis-related DEGs (CDEGs), disulfidptosis-related DEGs (DDEGs), ferroptosis-related DEGs (FDEGs), and pyroptosis-related DEGs (PDEGs).

The functional enrichment analyses of ADEGs, CDEGs, DDEGs, FDEGs, and PDEGs were conducted using the clusterProfiler package in the R software. These analyses were based on GO and KEGG databases.

### Signature genes identification

Through the analysis of DGEs related to cell death and key module genes, a set of potential hub genes were identified. Machine learning is a variety of algorithms that enable computer programs to learn from data or past experience and then optimize their performance^17^. These hub genes were further screened using two machine learning algorithms, including the least absolute shrinkage and selection operator (LASSO) using glmnet package^18^, and Random Forest analysis using randomForest package in R software^19^. The LASSO analysis, which incorporates penalty parameters and cross-validation, was chosen as it is a more effective method for evaluating high-dimensional data compared to traditional regression analysis. Additionally, the Random Forest was employed to classify the cell death related DGEs for the hub genes. The optimal number of variables was determined by calculating the average error rate and the optimal number of trees was determined based on the lowest error rate. Once these parameters were established, a Random Forest was constructed and the feature importance scores of each candidate hub gene were determined. Genes with Mean Decrease Gini Index greater than 0.25 were selected. The intersection of the results from the LASSO analysis and Random Forest identified the signature genes of keratoconus. The diagnostic efficiency of these signature genes was assessed using the area under the receiver operating characteristic curves (AUCs of ROCs) in both of training and validation sets. A favorable diagnostic performance was indicated by an AUC greater than 0.7.

### Gene set enrichment analysis

Based on the median value of hub gene expression, we categorized the keratoconus cohort and conducted gene set enrichment analysis (GSEA) to ascertain the correlation between these signature genes and signaling pathways across various subgroups (p < 0.05)^20^.

### Statistical analysis

All statistical analyses in the current study were conducted using R software (version 4.2.2). Unless otherwise specified, a significance level of P ≤ 0.05 was considered statistically significant, and all p values were two-tailed.

### Ethical approval

GEO belongs to public databases. The patients involved in the database have obtained ethical approval. Users can download relevant data for free for research and publish relevant articles. Our study is based on opensource data, so there are no ethical issues.

## Results

### Datasets characteristics

According to the established retrieval strategy, we searched the GEO databases. A total of 36 datasets were identified. Then, based on the inclusion criteria, two datasets were finally included. GSE151631, the training set, comprising 19 individuals with keratoconus and 7 control subjects. GSE77938, the validation group, consisting of 25 individuals with keratoconus and 25 control subjects. The participants information of the two datasets were summarized as Table 1, including the GEO ID, platform, sample size, definitions of the disease, stage of keratoconus, and ethnicities of participants (Table 1).

**Table 1:** The participants information of the two datasets.

ID	Platform	Sample size	Definitions of the disease	Stage of keratoconus	Ethnicities/country
GSE151631	GPL16791 Illumina HiSeq 2500	Keratoconus: 19	Keratoconus: central keratometry > 52 diopters (D) or not measurable	Severe degree	Keratoconus: African American
		Control: 5	Control: no keratoconus		Control: European American, Saudi Arabian and unknown
GSE77938	GPL18460 Illumina HiSeq 1500	Keratoconus: 25	Keratoconus: Patients had at least one clinical sign: corneal thinning, Vogt’s striae, or Fleischer rings	With surgical indications of. Keratoplasty (severe degree)	Pole
		Control: 25	Control: no keratoconus		

### Identification of DEGs between keratoconus and control and function enrichment analysis

**Figure 2:**
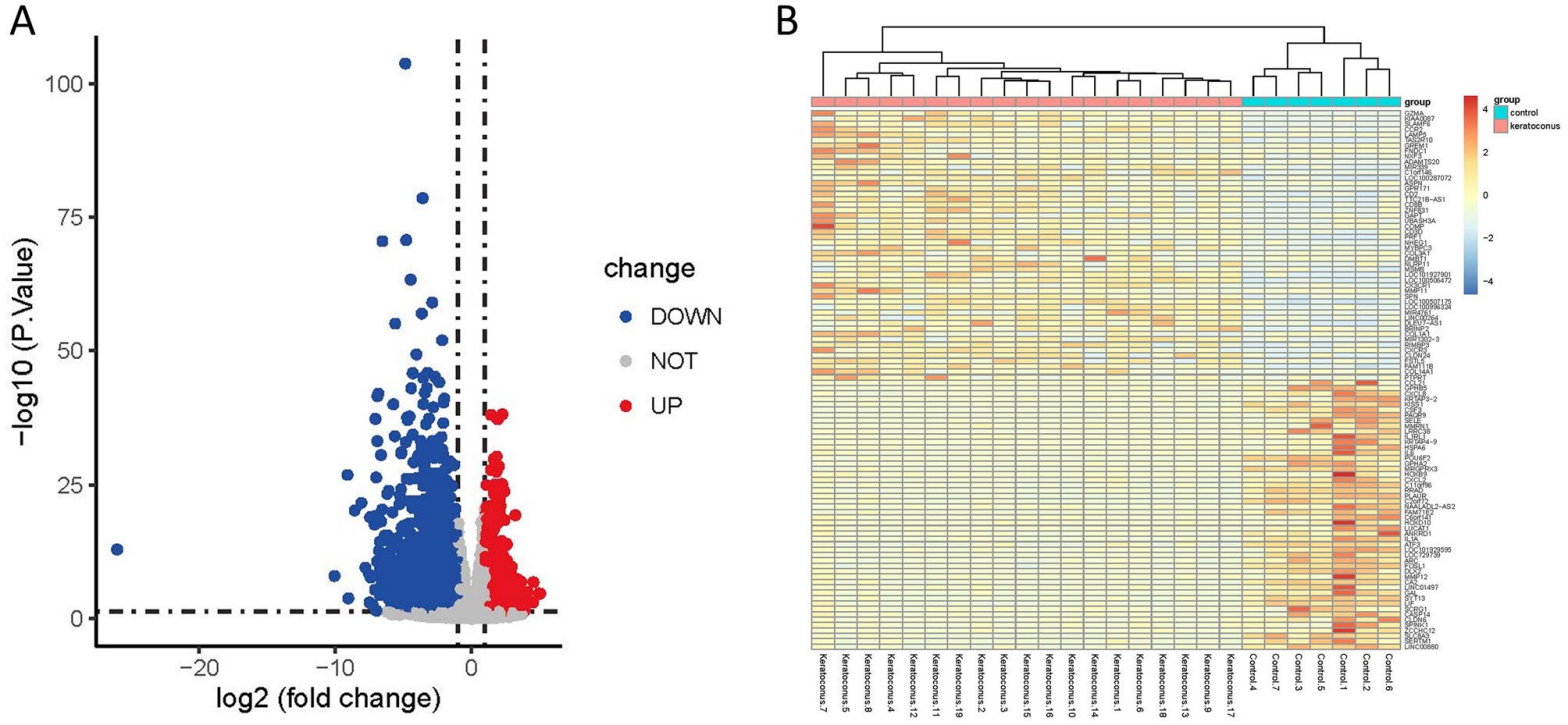
Identification of the DEGs in keratoconus. (**A**) Volcano showed expression of DEGs between the keratoconus and healthy cohort. (**B**) The heatmap showed the top 50 up-regulated DEGs and 50 down-regulated DEGs.

**Figure 3:**
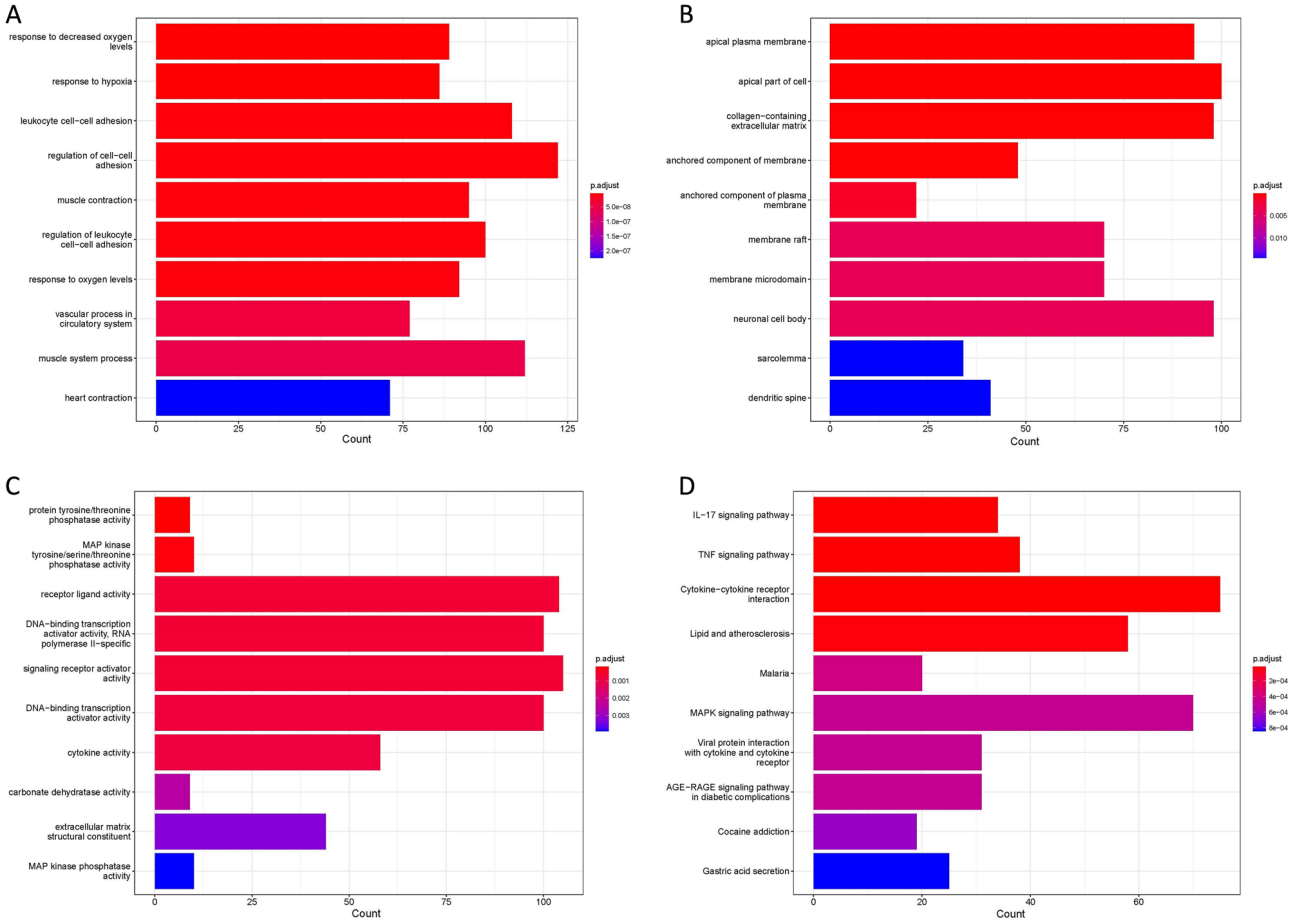
Functional enrichment analysis of DEGs in keratoconus. (**A**) The top 10 functional enrichment in BP. (**B**) The top 10 functional enrichment in CC. (**C**) The top 10 functional enrichment in MF. (**D**) The KEGG analysis of DEGs in keratoconus.

A total of 3558 DEGs were identified, consisting of 1154 up-regulated genes and 2404 down-regulated genes (Fig. 2A). To visualize the expression patterns of these DEGs, a heatmap was generated, displaying the top 50 up-regulated and top 50 down-regulated genes between the keratoconus group and the control group (Fig. 2B). The BP analysis revealed significant enrichment of processes such as response to decreased oxygen levels, response to hypoxia, leukocyte cell–cell adhesion, and regulation of cell–cell adhesion (Fig. 3A). In the CC analysis, the top three enriched components were the apical plasma membrane, apical part of the cell, and collagen-containing extracellular matrix (Fig. 3B). Furthermore, the MF analysis highlighted the importance of protein tyrosine/threonine phosphatase activity, MAP kinase tyrosine/serine/threonine phosphatase activity, and receptor ligand activity (Fig. 3C). Finally, the KEGG analysis identified the IL-17 signaling pathway, TNF signaling pathway, and cytokine-cytokine receptor interaction as the top three enriched pathways (Fig. 3D).

### Identification of ADEGs, CDEGs, DDEGs, FDEGs and PDEGs and function enrichment analysis

**Figure 4:**
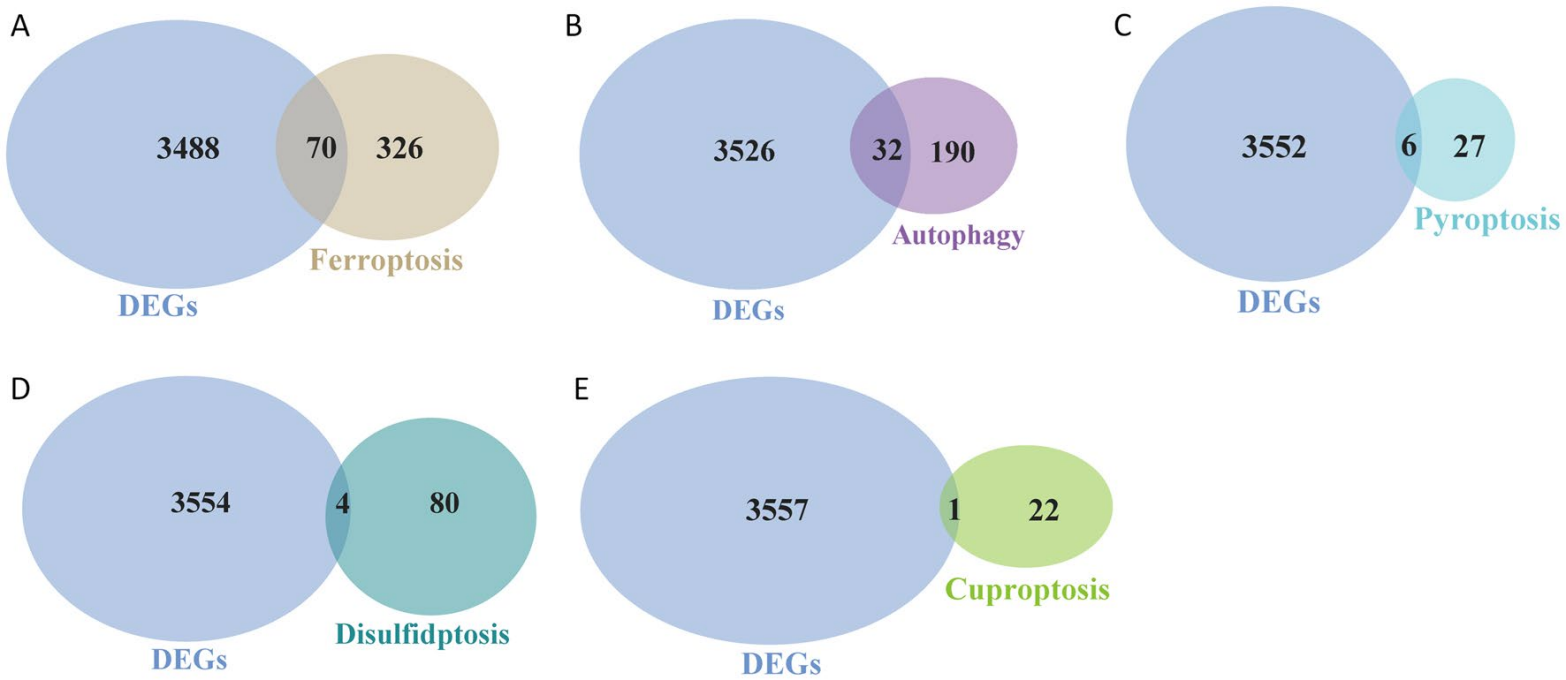
Identification of candidate hub genes. (**A**) The two Venn plot showed the interaction between DEGs and ferroptosis related genes dataset. (**B**) The two Venn plot showed the interaction between DEGs and autophagy related genes dataset. (**C**) The two Venn plot showed the interaction between DEGs and pyroptosis related genes dataset. (**D**) The two Venn plot showed the interaction between DEGs and disulfidptosis related genes dataset. (**E**) The two Venn plot showed the interaction between DEGs and cuproptosis related genes dataset.

To further identified ADEGs, CDEGs, DDEGs, FDEGs and PDEGs from keratoconus and control cornea, we took the intersections between the corresponding dataset and DEGs using the “DESeq2” package in the R software. We identified 70 FDEGs (Fig. 4A), 32 ADEGs (Fig. 4B), 6 PDEGs (Fig. 4C), 4 DDEGs (Fig. 4D) and 1 CDEGs (Fig. 4E).

**Figure 5:**
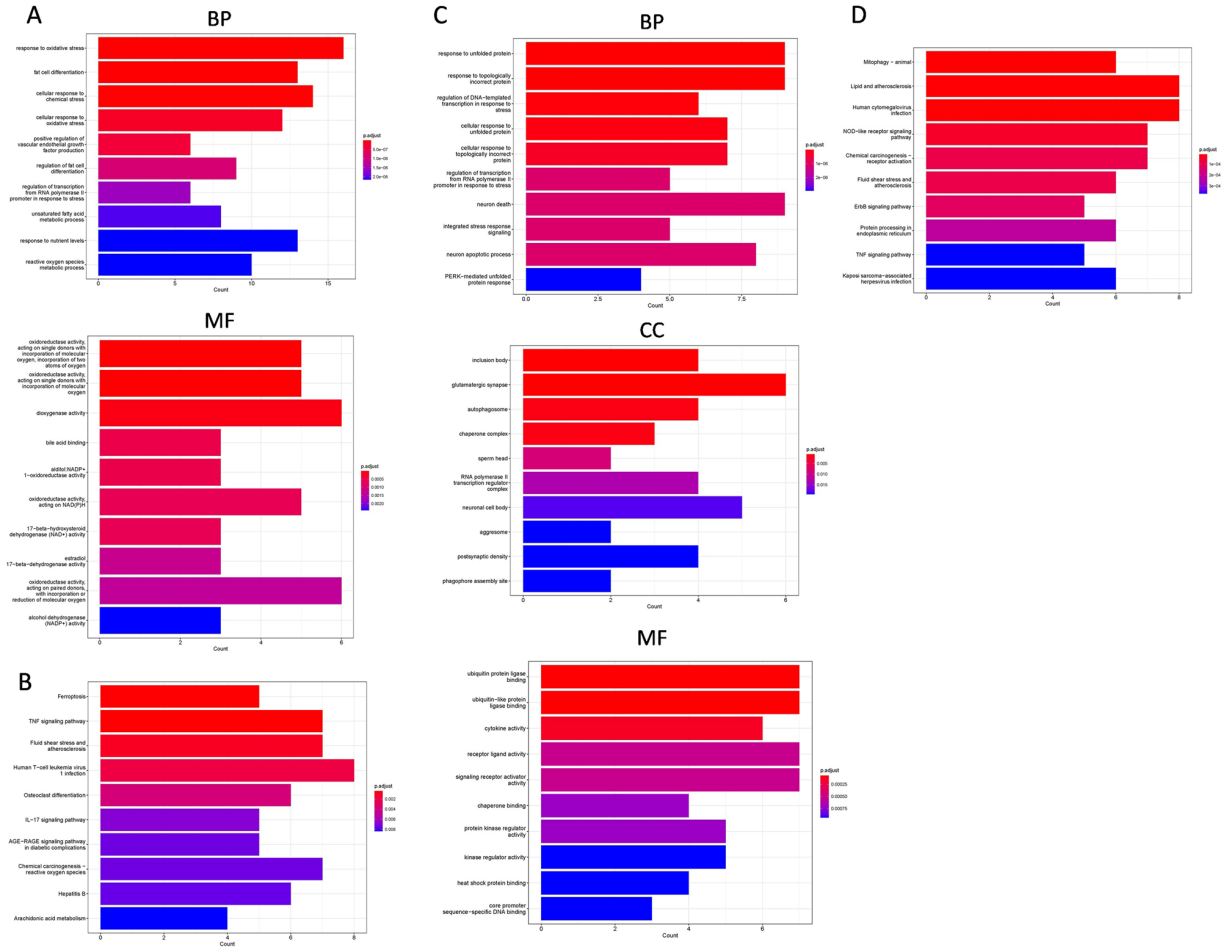
Functional enrichment analysis of FDEGs and ADEGs. (**A**) The top 10 functional enrichment in BP and MF of FDEGs. (**B**) The KEGG analysis of FDEGs. (**C**) The top 10 functional enrichment in BP, CC and MF of ADEGs. (**D**) The KEGG analysis of ADEGs.

Due to the restricted availability of CDEGs, DDEGs, and PDEGs, only FDEGs and ADEGs were incorporated into the examination. In the analysis of FDEGs, the BP observed that there was a significant enrichment in the response to oxidative stress (16 genes), fat cell differentiation (13 genes), and cellular response to chemical stress (14 genes) (Fig. 5A). Furthermore, oxidoreductase activity, dioxygenase activity, and bile acid binding were found to play a crucial role in the MF (Fig. 5A). However, due to a p value less than 0.05, the enrichment results for CC could not be obtained. The KEGG analysis revealed that the most enriched pathway was ferroptosis (5 genes), followed by the TNF signaling pathway (7 genes) and fluid shear stress and atherosclerosis (7 genes) (Fig. 5B).

The BP analysis of ADEGs revealed significant enrichment in response to unfolded protein (9 genes) and topologically incorrect protein (9 genes), as well as regulation of DNA-templated transcription in response to stress (6 genes) (Fig. 5C). In terms of CC, the top three enriched categories were inclusion body (4 genes), glutamatergic synapse (6 genes), and autophagosome (4 genes) (Fig. 5C). Furthermore, MF analysis highlighted the importance of ubiquitin protein ligase binding (7 genes), ubiquitin-like protein ligase binding (7 genes), and cytokine activity (6 genes) (Fig. 5C). The KEGG analysis identified the top three enriched pathways as mitophagy (animal) (6 genes), lipid and atherosclerosis (8 genes), and human cytomegalovirus infection (8 genes) (Fig. 5D).

### Selection of signature genes

We screened out signature FDEGs and ADEGs from candidate key genes in keratoconus by LASSO analysis and Random Forest analysis.

**Figure 6:**
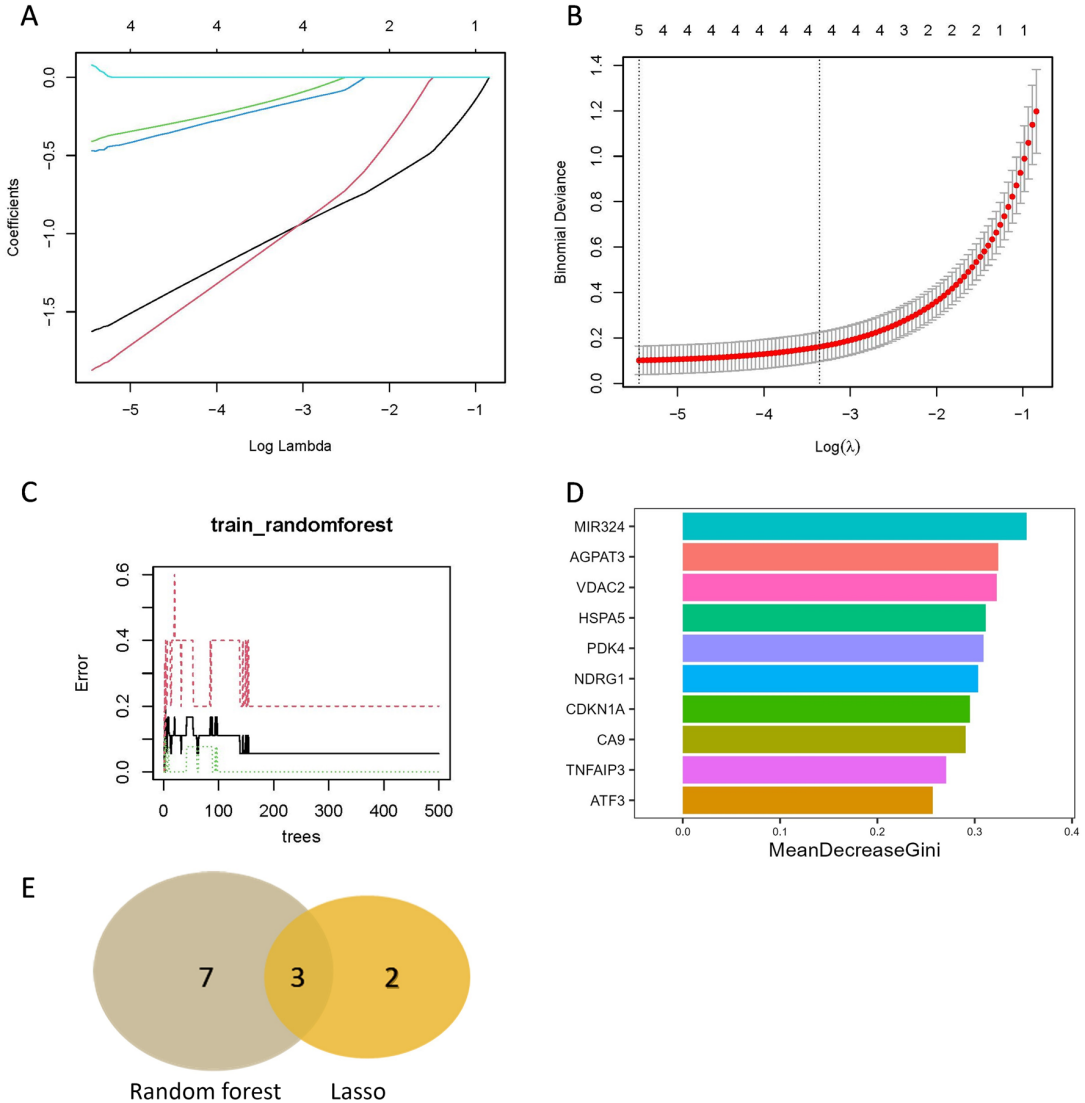
The machine algorithms for FDEGs. (**A**) LASSO plot showed the variations in the size of coefficients for parameters shrank as the value of k penalty increased. (**B**) Penalty plot of the LASSO with error bars denoting standard errors. (**C**) The error rate confidence intervals for Random Forest. (**D**) The relative importance of genes is more than 0.25 in Random Forest, (**E**) The interaction of the LASSO and Random Forest algorithms for signature FDEGs.

In the LASSO analysis, a total of five signature FDEGs were selected (Fig. 6A,B). On the other hand, the Random Forest analysis identified ten signature FDEGs with a relative importance greater than 0.25 (Fig. 6C,D). These signature genes can be found in Table 2. By considering the results from both algorithms, three signature genes were ultimately determined (Fig. 6E): 1-acylglycerol-3-phosphate O-acyltransferase 3 (AGPAT3), tumor necrosis factor alpha induced protein 3 (TNFAIP3), and carbonic anhydrase 9 (CA9).

**Table 2:** The key FDEGs screened out through LASSO and random forest analysis**.**

LASSO analysis	Random forest algorithms
TNFAIP3, MIR27A, CDO1, CA9, AGPAT3	ATF3, TNFAIP3, CA9, CDKN1A, NDRG1, PDK4, HSPA5, VDAC2, AGPAT3, MIR324

**Figure 7:**
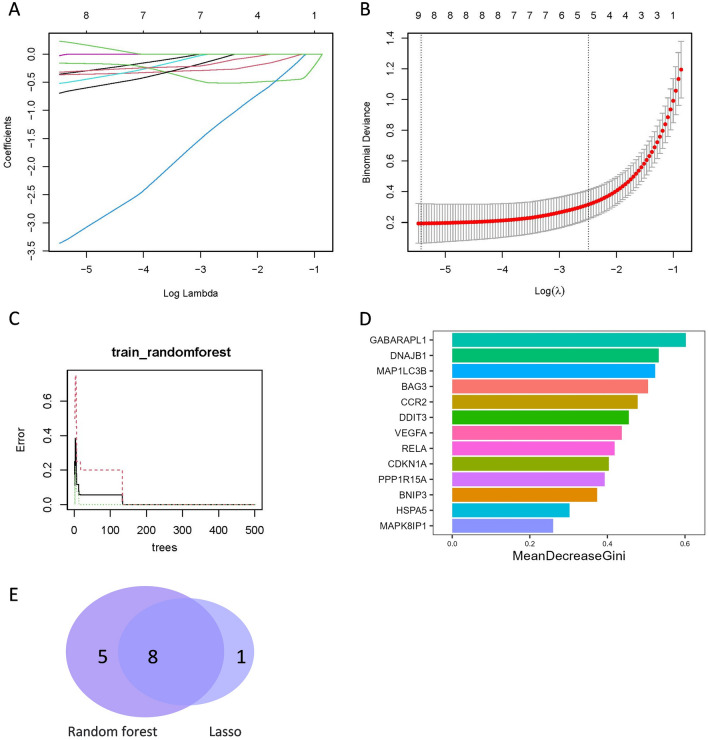
The machine algorithms for ADEGs. (**A**) LASSO plot showed the variations in the size of coefficients for parameters shrank as the value of k penalty increased. (**B**) Penalty plot of the LASSO with error bars denoting standard errors. (**C**) The error rate confidence intervals for Random Forest. (**D**) The relative importance of genes is more than 0.25 in Random Forest. (**E**) The interaction of the LASSO and Random Forest algorithms for signature ADEGs.

In the analysis of ADEGs, a total of nine signature ADEGs were identified using LASSO analysis (Fig. 7A,B). Additionally, the Random Forest analysis revealed thirteen signature ADEGs with a relative importance greater than 0.25 (Fig. 7C,D). These signature genes are listed in Table 3. By considering the results from both algorithms, a final set of eight signature genes was determined, namely vascular endothelial growth factor A (VEGFA), protein phosphatase 1 regulatory subunit 15A (PPP1R15A), mitogen-activated protein kinase 8 interacting protein 1 (MAPK8IP1), microtubule associated protein 1 light chain 3 beta (MAP1LC3B), heat shock protein family A (Hsp70) member 5 (HSPA5), DNA damage inducible transcript 3 (DDIT3), cyclin dependent kinase inhibitor 1A (CDKN1A), and C–C motif chemokine receptor 2 (CCR2) (Fig. 7E).

**Table 3:** The key ADEGs screened out through LASSO and random forest analysis.

LASSO analysis	Random forest algorithms
VEGFA, PPP1R15A, MAPK8IP1, MAP1LC3B, HSPA5, DNAJB9, DDIT3, CDKN1A, CCR2	CDKN1A, DDIT3, BNIP3, HSPA5, BAG3, GABARAPL1, RELA, MAP1LC3B, MAPK8IP1, DNAJB1, PPP1R15A, VEGFA, CCR2

### Diagnostic efficacy of signature genes in keratoconus

**Figure 8:**
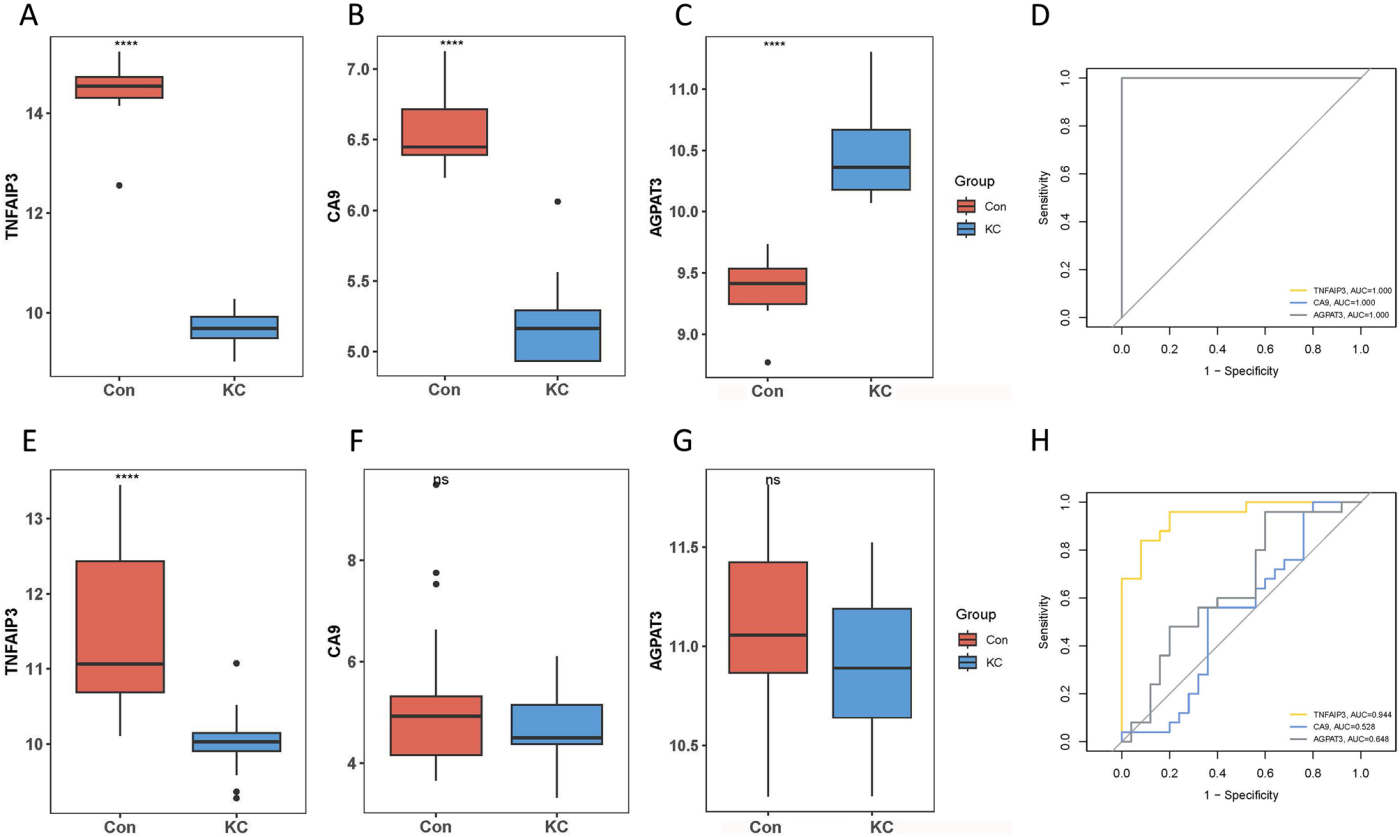
The performance of the FDEGs in test and validation cohort, respectively. (**A**–**C**) The expression of signature FDEGs between the keratoconus and control in test cohort. (**D**) ROC showed the diagnostic performance of the signature FDEGs in test cohort. (**E–G**) The expression of signature FDEGs between the keratoconus and control in validation cohort. (**H**) ROC showed the diagnostic performance of the signature FDEGs in validation cohort. "ns" means P > 0.05. *P ≤ 0.05. **P ≤ 0.01. ***P ≤ 0.001.and****P ≤ 0.0001.

For FDEGs, the expression of certain genes identified through screening was found to be significantly different compared to control group (P < 0.05). This suggests that these genes may have a potential role in the development of keratoconus (Fig. 8A–D). Subsequently, in an independent validation cohort, the diagnostic accuracy of these signature genes in keratoconus was assessed. Consistent with the findings from a previous study (GSE77938), only the expression of TNFAIP3 (Tumor necrosis factor alpha induced protein 3) was significantly decreased in individuals with keratoconus compared to control subject (Fig. 8E–H). In summary, these results indicate that TNFAIP3 may be a noteworthy gene associated with FDEGs, and it may have diagnostic utility in keratoconus.

**Figure 9:**
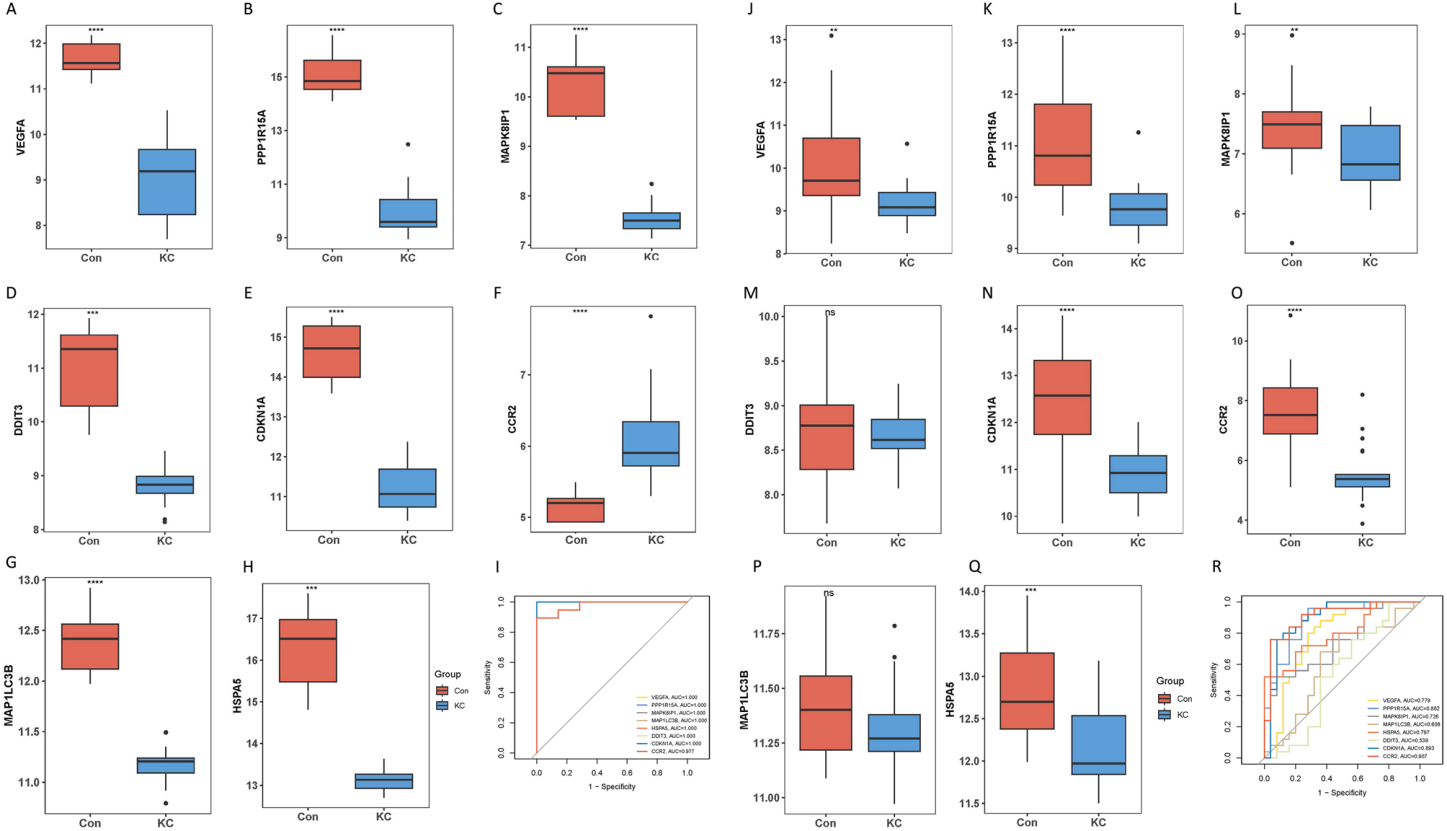
The performance of the ADEGs in test and validation cohort, respectively. (**A**–**H**) The expression of signature ADEGs between the keratoconus and control in test cohort. (**I**) ROC showed the diagnostic performance of the signature ADEGs in test cohort. (**J–Q**) The expression of signature ADEGs between the keratoconus and control in validation cohort. (**R**) ROC showed the diagnostic performance of the signature ADEGs in validation cohort. "ns" means P > 0.05. *P ≤ 0.05. **P ≤ 0.01. ***P ≤ 0.001.and****P ≤ 0.0001.

For ADEGs, the expression of the screened signature genes in individuals with keratoconus was found to be significantly different from that in control subjects (P < 0.05), suggesting a potential role of these genes in the development of keratoconus (Fig. 9A–I). Furthermore, the diagnostic efficiency of these signature genes in keratoconus was evaluated in GSE77938. The expression of CCR2, CDKN1A, HSPA5, MAPK8IP1, PPP1R15A, and VEGFA in individuals with keratoconus was significantly different from that in control subjects. However, it was observed that the expression of CCR2 was significantly reduced in individuals with keratoconus in the external validation cohort, while it was significantly increased in the test cohort (Fig. 9J–R).

### GSEA analysis

Based on the above results, we assessed signaling pathways associated with the signature FDEGs and ADEGs via GSEA analysis respectively.

**Figure 10:**
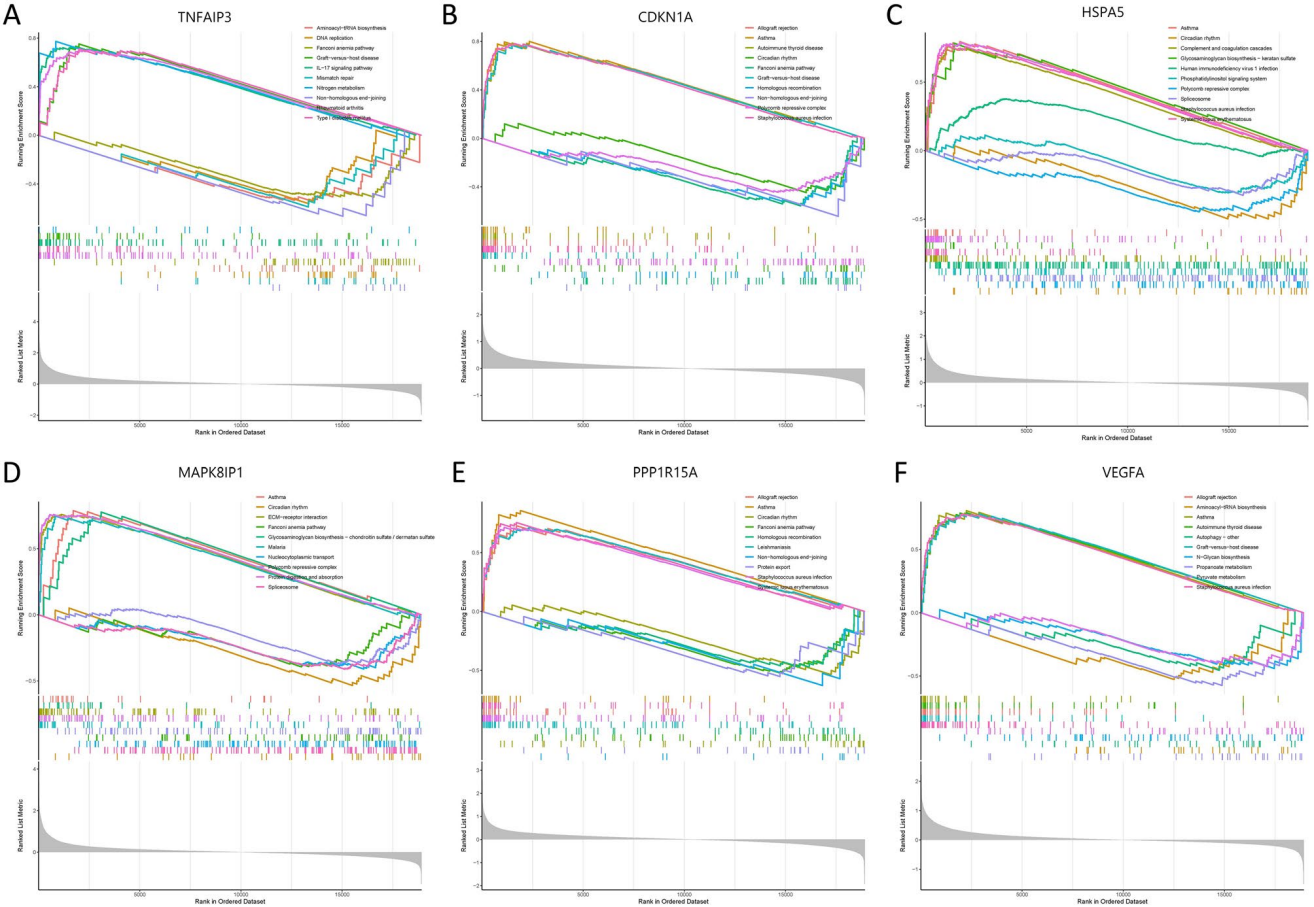
The GSEA of remarkable signature genes in keratoconus. (**A**) The GSEA of TNFAIP3 in keratoconus. (**B**) The GSEA of CDKN1A in keratoconus. (**C**) The GSEA of HSPA5 in keratoconus. (**D**) The GSEA of MAPK8IP1 in keratoconus. (**E**) The GSEA of PPP1R15A in keratoconus. (**F**) The GSEA of VEGFA in keratoconus.

For FDEGs, the results showed that TNFAIP3 was significantly correlated with aminoacyl-tRNA biosynthesis, DNA replication, Fanconi anemia pathway, graft-versus-host disease, IL-17 signaling pathway, mismatch repair, nitrogen metabolism, non-homologous end-joining, rheumatoid arthritis, type I diabetes mellitus. In summary, these genes all positively correlated with gene replication and mutation, immune response, and energy metabolism signaling pathway (Fig. 10A).

For ADEGs, the results showed that CDKN1A was significantly correlated with allograft rejection, asthma, autoimmune thyroid disease, circadian rhythm, Fanconi anemia pathway, graft-versus-host disease, homologous recombination, non-homologous end-joining, polycomb repressive complex, staphylococcus aureus infection (Fig. 10B). HSPA5 significantly correlated with asthma, circadian rhythm, complement and coagulation, glycosaminoglycan biosynthesis-keratan sulfate, human immunodeficiency virus 1 infection, phosphatidylinositol signaling system, polycomb repressive complex, spliceosome, staphylococcus aureus infection, and systemic lupus erythematosus (Fig. 10C). MAPK8IP1 significantly correlated with asthma, circadian rhythm, ECM-receptor interaction, Fanconi anemia pathway, glycosaminoglycan biosynthesis-chondroitin sulfate/dermatan sulfate, malaria, nucleocytoplasmic transport, polycomb repressive complex, protein digestion and absorption, and spliceosome (Fig. 10D). PPP1R15A significantly correlated with allograft rejection, asthma, circadian rhythm, Fanconi anemia pathway, homologous end-joining, protein export, staphylococcus aureus infection, and systemic lupus erythematosus (Fig. 10E). VEGFA significantly correlated with allograft rejection, aminoacyl-tRNA biosynthesis, asthma, autoimmune thyroid disease, autophagy-other, graft-versus-host disease, N-Glycan biosynthesis, propanoate metabolism, pyruvate metabolism, and staphylococcus aureus infection (Fig. 10F). In summary, these genes all mainly correlated with autoimmune disease, pyruvate metabolism, and microbial infection.

## Discussion

The latest epidemiological studies have revealed that the global incidence of keratoconus ranges from 0.2 to 4790 per 100,000 individuals, with the highest incidence observed among individuals aged 20–30 years^21,22^. This high incidence highlights keratoconus as a common cause of vision impairment. Current clinical treatments for keratoconus, such as rigid gas permeable contact lenses (RGP) and corneal collagen cross-linking, aim to control the progression of the disease^23^. However, some cases still worsen after treatment, leading to corneal perforation. However, underlying causes and mechanisms of keratoconus, particularly the key disease genes involved, remain unclear. Consequently, developing effective treatment strategies and addressing the problem at its root have proven challenging. This study aimed to identify DEGs between keratoconus and control cohorts, resulting in the identification of one signature FDEG and five signature ADEGs. These six genes hold significant value in furthering our understanding of the etiology and development mechanisms of keratoconus.

Two databases, GSE151631 and GSE77938, were included in this study based on the established retrieval strategy and the inclusion criteria. The description of the diagnostic criteria for keratoconus is slightly different in the two datasets. However, all of the keratoconus samples in these two datasets were derived from keratoconus patients who had the surgical indications for keratoplasty. It might imply that all patients of these two datasets might all be diagnosed at the late stage of keratoconus. In addition, previous researches demonstrated that the incidence of keratoconus may differ among different regions and ethnicities^24^. Asian groups might have a significantly higher prevalence rate than that of whites^25^. In this study, there are no Asian keratoconus patients in both two datasets. In the future, more similar research and datasets (particularly including Asian keratoconus patients) would be expected to be published and verify our results.

Besides DESeq2 package, there are two other R software packages, LIMMA and edgeR, are the most known packages for applying the differential expression analysis. However, there are some differences among the three methods. The main differences are summarized as follows: (1) Both DESeq2 and edgeR are popular choice for gene discovery through the differential expression analysis for RNA-sequencing data, while LIMMA are suitable for microarrays, RNA-sequencing and quantitative PCR^26^; (2) The normalized RNA-sequencing count data is necessary for edgeR and LIMMA, whereas DESeq2 uses its own library discrepancies to correct data instead of normalization^13^; (3) Based on different statistical methodologies, DESeq2 and edgeR obtain more DEGs than LIMMA^27^. Therefore, the DESeq2 was selected in this study.

Functional enrichment analyses were conducted on the DEGs identified in this study. The results revealed that these DEGs were associated with response to hypoxia, regulation of cell–cell adhesion, protein tyrosine/threonine phosphatase activity, and the activity of the mitogen-activated protein kinases (MAPK) signaling pathway, receptor ligand activity played essential. The top three enriched pathways associated with these DEGs were the IL-17 signaling pathway, TNF signaling pathway, and cytokine-cytokine receptor interaction. These findings are consistent with previous studies which demonstrated that inflammatory cytokines and collagen degradation have been implicated in the pathological mechanisms of keratoconus^28–31^. For instance, the research of Lema et al. suggested that the levels of pro-inflammatory cytokines IL-6, TNF-α, and matrix metalloproteinase 9 (MMP-9) of tear film in keratoconus significantly increased^32^. Additionally, as the cornea is directly exposed to solar ultraviolet radiation, which can lead to oxidative stress injury due to excessive free radicals from atmospheric pollution and oxygen, including reactive oxygen species (ROS)^33^. Research has shown that oxidative stress markers are elevated and antioxidants are decreased in keratoconus samples compared to healthy individuals^34^. Similarly, the antioxidant capacity and glutathione content in keratoconus corneas were significantly reduced compared to healthy corneas^35^. Overall, these findings suggest that abnormalities in these DEGs may contribute to the pathological changes observed in keratoconus.

The excessive production of ROS can have detrimental effects on cellular structures, such as the peroxidation of lipids in cell membranes, damage to mitochondrial DNA, and the initiation of cell apoptosis through autophagy and further lead to corneal diseases including keratoconus^36^. A study found that keratocytes derived from keratoconus cases showed increased expression of proteins related to apoptosis and endocytosis, suggesting degeneration and subsequent apoptosis of these resident stromal cells^37^. Autophagy is a self-regulated process in which cytoplasmic proteins or organelles are engulfed and enclosed in vesicles, which then fuse with lysosomes to form autophagic lysosomes. The contents of these lysosomes are degraded, allowing for cellular metabolism and organelle renewal^38^. Autophagy and apoptosis are closely related processes in cell death. Research has shown that defects in autophagy can lead to the accumulation of non-degradable cellular materials or organelle deposits in keratoconus^39^. Oxidative stress-induced defects in the autophagy-lysosomal pathway have been suggested to be involved in the progression and pathogenesis of keratoconus^40^. Further researches have revealed various forms of cell death, such as ferroptosis, cuproptosis, and disulfidptosis, which are believed to contribute to the development of different diseases. In this study, we identified 70 FDEGs, 32 ADEGs, 6 PDEGs, 4 DDEGs, and 1 CDEG through intersection analysis of datasets. Due to the limited number of CDEGs, DDEGs, and PDEGs, only FDEGs and ADEGs were included in the analysis as hub genes. Interestingly, the top 10 results of Go and KEGG analyses showed significant overlap between the 3558 DEGs, 70 FDEGs (1.97% of all DEGs) and the 32 ADEGs (0.90% of all DEGs). These shared results included pathways such as the TNF signaling pathway, IL-17 signaling pathway, lipid metabolism, and atherosclerosis. This suggests that FDEGs and ADEGs, as well as the regulation of ferroptosis and autophagy by these genes, may play a significant role in the pathological process of keratoconus. These findings provide potential avenues for further research in this field.

In this study, two machine learning algorithms, LASSO and Random Forest, were employed to identify signature genes. As mentioned above, machine learning including a variety of statistical, probabilistic and optimization methods have a wide range of applications. LASSO is a popular machine learning method used for regression analysis. It improves the accuracy and interpretability of the result via variable selection and LASSO regularization. LASSO regularization also known as L1 regularization which is one of the models that shrinks regression coefficients to zero^41^. Random Forest is also a popular machine learning method. It aims at forecasting continuous variables and delivering prediction outcomes with minimal observable variability. The technique has the advantages of being unaffected by variable conditions and achieving higher accuracy, sensitivity and specificity^42^. Based on these characteristic, LASSO and Random Forest analysis are widely applied for selecting signature genes of various diseases. For the same reason, the present study applied LASSO and Random Forest to pick out signature genes (ADEGs and FDEGs) from DEGs. These results of this study suggest that one FDEGs (TNFAIP3) and five ADEGs (CDKN1A, HSPA5, MAPK8IP1, PPP1R15A, and VEGFA) may be noteworthy differentially expressed genes associated with keratoconus, and they have potential diagnostic efficiency in the disease. For ADEGs, Go and KEGG pathways demonstrated that these aberrantly expressed genes were not only enriched in autophagy, but also in other cellular functions and processes. CDKN1A, also known as CDKN1 or P21, encodes a protein that interacts with proliferating cell nuclear antigen, a DNA polymerase accessory factor. It plays a regulatory role in the synthesis phase of DNA replication and DNA damage repair^43^. Previous studies have shown that CDKN1A may be involved in apoptosis following caspase activation and tissue regeneration in mice lacking the CDKN1A gene^44,45^. Additionally, CDKN1A has been found to be increased in corneal epithelial cells undergoing replicative senescence and in corneal epithelial cells cultured from older donors^46,47^. Furthermore, enhanced nuclear expression of CDKN1A has been observed in the endothelium of Fuchs endothelial corneal dystrophy^48^. The findings of this study indicate that the expression of CDKN1A is decreased in keratoconus compared to control corneas, suggesting impairment in the processes of DNA replication and DNA damage repair in keratoconus.

HSPA5, a member of the heat shock protein family A (Hsp70), is primarily located in the endoplasmic reticulum (ER) and functions as a key regulator of ER homeostasis by suppressing the unfolded protein response^49^. Additionally, it is involved in cellular apoptosis and senescence. Previous studies have established a connection between ER stress and various corneal diseases^50,51^. Our research findings demonstrate a decrease in HSPA5 expression in keratoconus compared to control corneas, suggesting that ER stress may contribute to the susceptibility of keratoconus. The genes MAPK8IP1, PPP1R15A, and VEGFA are associated with cellular inflammation and apoptosis.

Ferroptosis is a distinct form of cell death induced by oxidative stress and characterized by iron dependency^52^. Numerous studies have linked ferroptosis to oxidative stress^53,54^, which is recognized as a risk factor for keratoconus^34,55^. However, the interplay between oxidative stress and ferroptosis in the pathogenesis of keratoconus remains unclear. TNFAIP3, also known as A20, has been shown to inhibit NF-κB activation and TNF-mediated apoptosis, and it also plays a role in cytokine-mediated immune and inflammatory responses^56^. In the context of the ocular surface, TNFAIP3 has been identified as a potent regulator of the corneal epithelium's response to inflammation in dry eye^57^.

Cuproptosis refers to a form of cell death that is dependent on the presence of copper^58^. On the other hand, disulfidptosis is characterized by the accumulation of disulfides, which leads to disulfide stress and subsequent cell death^11^. However, due to the limited number of DDEGs and CDEGs in our study, we were unable to perform LASSO analysis and Random Forest analysis. Therefore, further research is needed to explore and analyze these factors in future studies.

The GSEA results revealed that the signature FDEGs and ADEGs were primarily associated with autoimmune diseases, immune system impairment, inflammation, substance metabolism, and genetic information synthesis. This finding aligns with the current understanding of the etiology and pathogenesis of keratoconus. Previous studies have reported a positive association between allergy, atopy, and keratoconus, with reported prevalence rates ranging from 11 to 30%^59^. When considering our study's results, it is possible that the immune system abnormalities observed in keratoconus may be linked to processes such as autophagy and ferroptosis. Although keratoconus is traditionally classified as a non-inflammatory disease, emerging evidence suggests the presence of certain inflammatory characteristics^28^. This may be indicative of immune system abnormalities in keratoconus.

## Conclusions

In brief, this study identified six genes that are associated with ferroptosis and autophagy, namely TNFAIP3, CDKN1A, HSPA5, MAPK8IP1, PPP1R15A, and VEGFA. These genes have significant relevance in investigating the causes and progression of keratoconus. The findings of this study have important implications for understanding the pathology of keratoconus, both in terms of theoretical knowledge and potential clinical applications. Additionally, these genes have been found to be linked to immune system disorders, suggesting new avenues for further research and treatment of keratoconus.

## Data Availability

The datasets generated during and/or analysed during the current study are available from the corresponding author on reasonable request.
